# Asymmetric analysis reveals novel virus capsid features

**DOI:** 10.1007/s12551-019-00572-9

**Published:** 2019-07-24

**Authors:** M. J. Conley, D. Bhella

**Affiliations:** MRC-University of Glasgow Centre for Virus Research, Institute of Infection, Immunity and Inflammation, College of Medical Veterinary and Life Sciences, Garscube Estate, University of Glasgow, Glasgow, Scotland G61 1QH UK

**Keywords:** Cryo-electron microscopy, Asymmetry, Virus, Capsid, Portal

## Abstract

Cryo-electron microscopy and single-particle image analysis are frequently used methods for macromolecular structure determination. Conventional single-particle analysis, however, usually takes advantage of inherent sample symmetries which assist in the calculation of the structure of interest (such as viruses). Many viruses assemble an icosahedral capsid and often icosahedral symmetry is applied during structure determination. Symmetry imposition, however, results in the loss of asymmetric features of the virus. Here, we provide a brief overview of the methods used to investigate non-symmetric capsid features. These include the recently developed focussed classification as well as more conventional methods which simply do not impose any symmetry. Asymmetric single-particle image analysis can reveal novel aspects of virus structure. For example, the VP4 capsid spike of rotavirus is only present at partial occupancy, the bacteriophage MS2 capsid contains a single copy of a maturation protein and some viruses also encode portals or portal-like assemblies for the packaging and/or release of their genome upon infection. Advances in single-particle image reconstruction methods now permit novel discoveries from previous single-particle data sets which are expanding our understanding of fundamental aspects of virus biology such as viral entry and egress.

## Introduction

Transmission electron microscopy has been widely used for the visualisation of biological macromolecules, including viruses, for several decades. In 1959, the now widely used method of negative staining was first described (Brenner and Horne 1959). Embedding macromolecular assemblies in a layer of heavy-metal salt provides excellent image contrast for sample evaluation, but is not suited to high-resolution structure determination. The demonstration of imaging frozen hydrated specimens at low temperature in the transmission electron microscope (cryoEM) in 1984 (Adrian et al. 1984) led to the emergence of a new field in structural biology. CryoEM permits the faithful imaging of biological molecules in a close-to-native state. Images may then be processed computationally to extract 3D structure information. For much of its history, cryoEM yielded 3D reconstructions at modest resolution and was not able to provide reliable information at the atomic level. Recent technological advances have, however, resulted in a resolution capability that rivals that of X-ray crystallography leading to the calculation of maps with very well-defined features, such as the recently solved structure of human apo-ferritin that was determined at 1.65Å resolution (Adrian et al. 1984; Zivanov et al. 2018). This advance in resolution of structures solved using cryo-electron microscopy (cryoEM) is largely due to developments such as direct detection devices and full automation of data collection in microscopes that may be kept cold for many days at a time. Software advances have likewise been important in extracting maximum information from image data. The field is developing further with the use of cold field emission guns which have already resulted in a 1.54Å structure of apo-ferritin (accession no. EMPIAR-10248), the highest resolution achieved to date using cryoEM (Iudin et al. 2016).

Single-particle analysis (SPA) is a commonly used method for determining the structure of a protein or macromolecular complex by cryoEM. This often involves imposing symmetry on the object of interest i.e. icosahedral symmetry on many virions and viral capsids. This allows the user to take advantage of inherent sample regularities/repeating units to assist in the structure determination process. Whilst this has proven to be a reliable and successful method of 3D reconstruction, it results in the loss of irregular, non-symmetric structural features which may be present in the object.

Probing asymmetry in objects that exhibit inherent symmetry can be challenging if the asymmetric feature is small or presents weak signal. In many cases, performing single-particle reconstruction without imposing symmetry, will nonetheless lead to a perfectly symmetric structure, owing to the dominating effect of strong symmetric features. A notable exception is that of the tailed bacteriophages, where a large tail assembly guarantees correct alignment of the asymmetric object (Tang et al. 2010). Asymmetric or ‘relaxed symmetry’ methods have yielded many informative structures at intermediate resolution in such cases.

The recent adoption of novel asymmetric 3D analysis techniques (of cryoEM SPA data sets) has resulted in a number of significant virological findings concerning smaller asymmetric features including portal-like assemblies and unique capsid proteins. Such studies are critical in expanding our understanding of key events in virus biology, such as morphogenesis and cell entry. Here, we review some recent studies, showing how asymmetric reconstruction methods have been used to identify novel or unexpected features of viruses.

## Eccentrically positioned capsids within enveloped virions

**Fig. 1:**
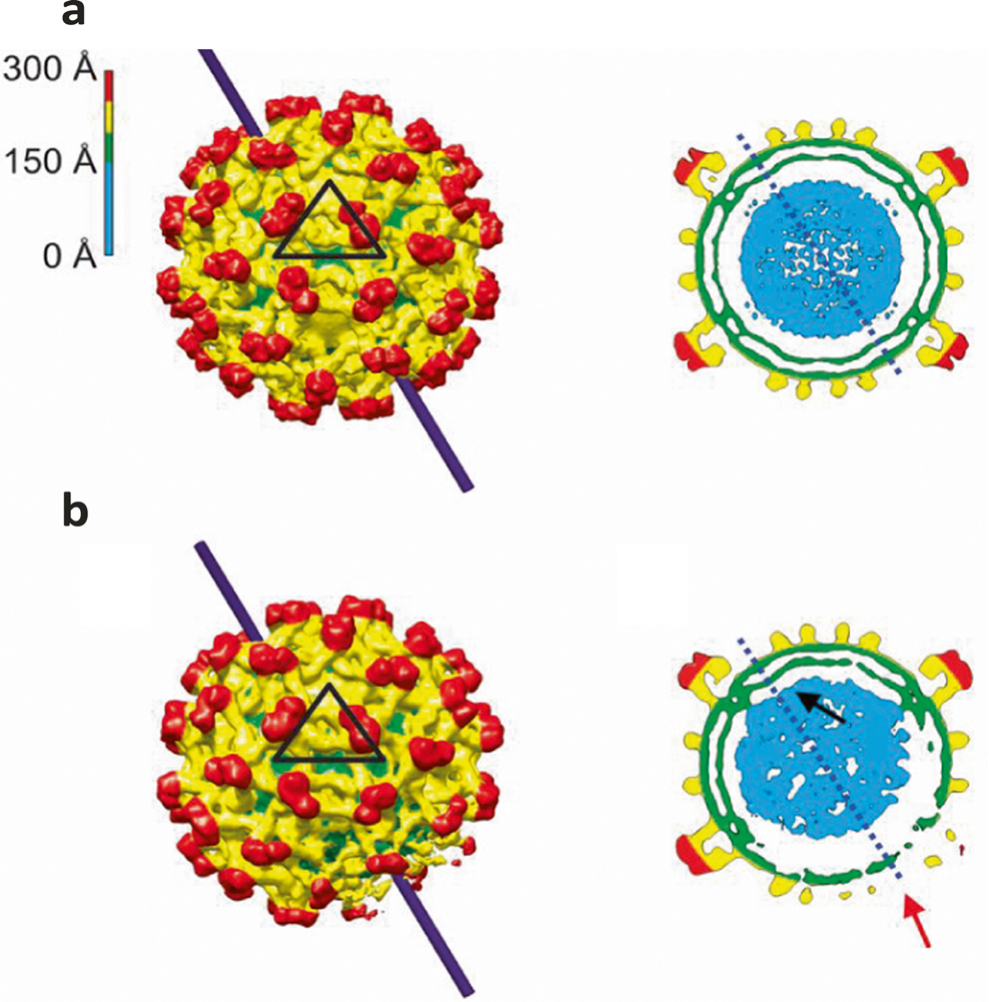
Asymmetry in immature capsids. Surface representations of KUNV (**a**; calculated with icosahedral symmetry and **b**; calculated without symmetry) as well as cross-sections through the structures illustrating the off-centre core particles (blue) which can only be visualised using asymmetric methods of image analysis. Image adapted from Therkelsen et al. 2018

Kunjin Virus (KUNV; a strain of West Nile Virus) is an enveloped, positive-sense RNA virus classified within the *Flaviviridae.* Viral maturation of KUNV occurs in the trans-golgi network leading to dramatic structural rearrangements in the virion. Immature KUNV virions contain 60 glycoprotein trimers (Zhang et al. 2003). Exposure to the low-pH environment of the trans-golgi network results in rearrangement of the 60 glycoprotein trimers into 90 dimers (Yu et al. 2008; Li et al. 2008). Flavivirus core particles, however, are only visible as a spherical shell with no regular structure (Kuhn et al. 2002). Structural studies suggest that the core associates with the viral membrane on the capsid exterior whilst associating with the genome at the interior (Ma et al. 2004). Asymmetric reconstruction (no symmetry imposed) was adopted to investigate the viral core in KUNV particles. Icosahedral and asymmetric reconstructions were performed to solve the structure of both immature (see Fig. 1) and mature KUNV virions. Both of the icosahedrally averaged structures showed a centred virus core. However, upon analysis with no symmetry imposed, the mature virions showed a centred core whilst the immature virions presented a core that was off-centre (Fig. 1b), supporting the previous hypothesis of the virion core associating with the viral membrane at one side only (Therkelsen et al. 2018).

## Visualising virus-membrane interactions

Picornaviruses are non-enveloped, positive-sense RNA viruses which enter host cells via endocytosis through the formation of ‘intermediate’ particles (Guttman and Baltimore 1977). Picornaviruses exhibit *T* = 1 and pseudo *T* = 3 symmetry with the capsids composed of VP1, VP2, VP3 and VP4 proteins which together form the 60 repeating structural units that assemble into the capsid structure (Muckelbauer et al. 1995). A picornavirus, Coxsackievirus B3 (CVB3), has been shown to form an entry intermediate (termed the A-particle) during the early stages of infection (Milstone et al. 2005). Lee et al. used nanodisc technology to mimic the receptor decorated membrane encountered by the virus during infection of host cells and imaged the nanodisc-bound particles using cryoEM followed by both icosahedral and asymmetric reconstruction methods (Lee et al. 2016). A pore was visualised in the CVB3 A-particle in the location adjacent to the nanodisc at a unique three-fold axis, representing the asymmetric features in this region. The weak density/pore in this region was described as being due to the flexible extrusions from capsid proteins VP1, VP2 and VP4. These protein extrusions were only visible at this location adjacent to the nanodisc and could only be visualised after determining the orientation of the particles using icosahedral symmetry followed by the relaxation of this symmetry to generate an asymmetric reconstruction of the CVB3 A-particle (Lee et al. 2016).

## Resolving features with partial/low occupancy

**Fig. 2:**
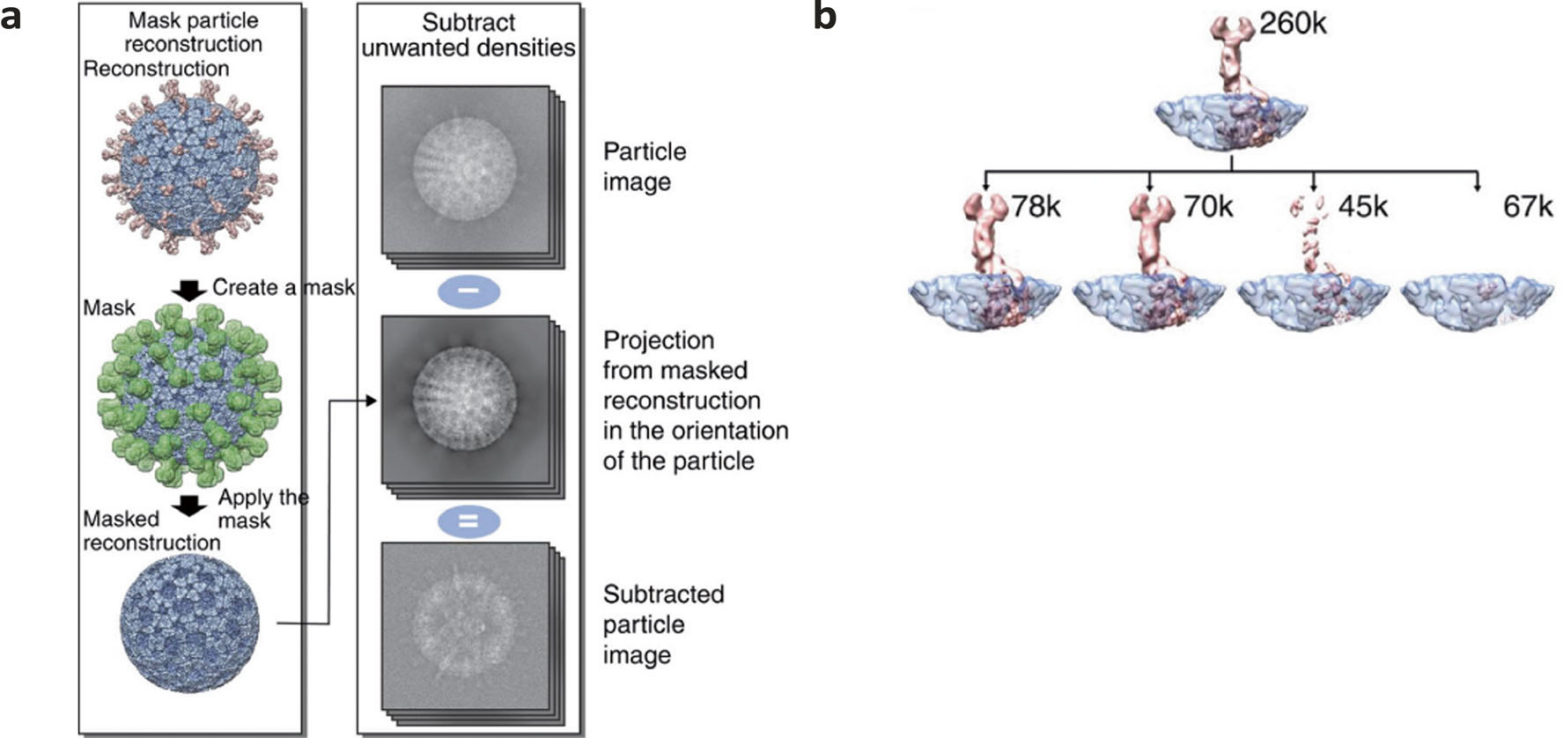
Density subtraction to investigate partial occupancy of the rotavirus VP4 protein. The masking of densities of interest followed by their subtraction to form a masked reconstruction and subsequent subtraction of this core reconstruction density from particle images is illustrated in panel **a**. This method of image analysis was used to separate 4 classes of rotavirus VP4 spike proteins from virions (**b**). The number of subparticles within each class is specified. As one class contained no density, this method allows the presence of partial occupancy to be investigated. Images taken and excerpted from Ilca et al. 2015 under a creative commons attribution 4.0 international licence

Rotaviruses are classified in the *Reoviridae* and are non-enveloped viruses containing a segmented double-stranded RNA genome. Rotaviruses are only able to infect vertebrates and are spread via the faecal-oral route (Rodriguez et al. 2014). Virions of rotaviruses are unusual in that they form a triple-layered particle (TLP) which surrounds the inner capsid (*T* = 1; formed of VP2 proteins). The TLP exhibits *T* = 13 icosahedral symmetry formed from 260 VP6 protein trimers which encapsidate the inner core (Settembre et al. 2011). The VP4 protein decorates the outer surface of the TLP with approximately 60 copies per virion although in the high-resolution structure of rotavirus, these features were poorly resolved (Settembre et al. 2011). This lower resolution of the VP4 spikes was hypothesised to be a consequence of partial occupancy or heterogeneity. Localised reconstruction methods were employed to address this. Briefly, a mask was applied to all of the 60 VP4 spikes in the full icosahedral TLP structure and this density was subtracted from the reconstruction. The TLP structure containing no VP4 spikes was then used to subtract capsid density from particle images (see Fig. 2a), leaving only the VP4 spike densities which were then subjected to 3D classification in Relion. This method showed 4 different classes of VP4 spike proteins; 2 presented clear VP4 density, 1 showed incomplete density whilst 1 class had no VP4 density (see Fig. 2b). Based on these data, the TLP appears to have only partial occupancy of the VP4 spikes, estimated at approximately 60–70% (Ilca et al. 2015). This partial occupancy of capsid proteins in virions was also observed for Kelp fly virus (KFV) where a turret-like structure was found to only occupy 29% of the five-fold vertices in the capsid (Briggs et al. 2005).

## Visualising low copy number proteins and viral genomes

**Fig. 3:**
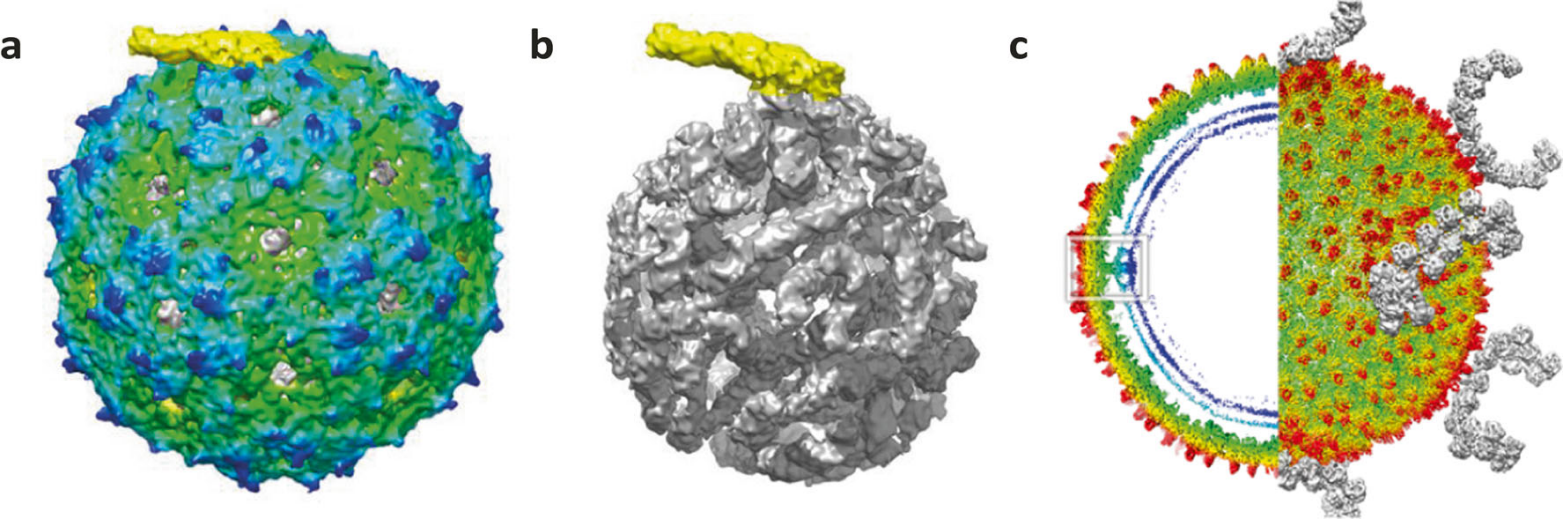
Identification of non-icosahedral capsid proteins. Bacteriophage MS2 (**a** and **b**) encodes a single A-protein per virus particle (yellow) which can be seen projecting out from the capsid surface (**a**) as well as interacting with the viral genome (grey; **b**). The archaeal SH1 virus (radially coloured in **c**) encodes horn-like capsid proteins (grey) on its surface which exhibit 2-fold symmetry. A 5-fold vertex structure is also highlighted with a white box (**c**). Images adapted from Koning et al. 2016 and De Colibus et al. 2019 under a creative commons attribution 4.0 international licence

Bacteriophages MS2 and Qβ are members of the *Leviviridae* and assemble a *T* = 3 icosahedral capsid that incorporates a single copy of the maturation/A-protein, which is responsible for binding both viral RNA and host receptors (Koning et al. 2016). The coat proteins form dimers that exhibit two slightly different conformations, A/B and C/C. The A-protein replaces one C/C capsomere at a single two-fold symmetry axis in the virion; thus, the capsid is formed of 178 coat proteins and 1 A-protein. Figure 3a shows the asymmetric reconstruction of the bacteriophage MS2 capsid with the unique A-protein highlighted in yellow. The use of 3D refinement without imposing any symmetry also revealed the ordered structure of the viral genome (grey) and how it interacts with the A-protein (yellow; Fig. 3b) (Koning et al. 2016). Similar findings have been reported for Qβ (Koning et al. 2016; Gorzelnik et al. 2016).

## Using localised reconstruction to resolve symmetry mismatches

The archaeal virus known as SH1 infects Haloarcula hispanica and has a double-stranded DNA genome which is encapsidated inside an internal membrane that is surrounded by a *T* = 28 icosahedral shell (Jaalinoja et al. 2008). SH1 virions are approx. 100 nm in diameter and are composed of capsid hexamers, decorated with 2 or 3 turret structures. Additionally, horn-like spikes with 2-fold symmetry are present at the 5-fold symmetry axes of the virion (Jaalinoja et al. 2008). Colibus et al. used a combination of asymmetric reconstruction (no symmetry imposed) followed by the localised reconstruction method described by Ilca et al. (see Fig. 2a) with 2-fold symmetry to resolve the virion and the horn-like spikes on the surface, respectively (see Fig. 3c) (De Colibus et al. 2019, Ilca et al. 2015).

## Structure determination of *in virio* macromolecular complexes

**Fig. 4:**
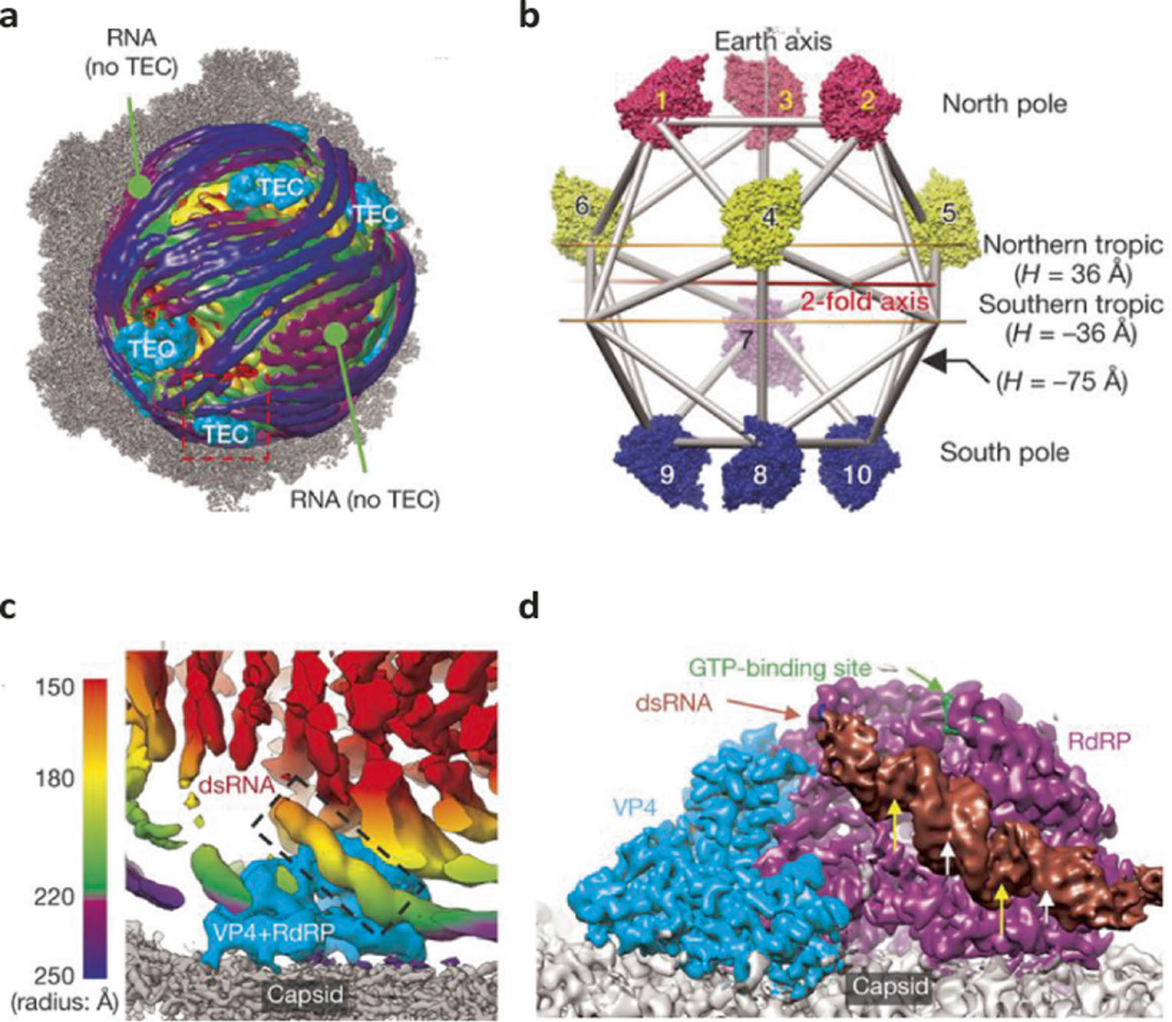
Transcriptional enzyme complexes (TECs) within the CPV virion. The CPV RNA genome and TECs (coloured) are shown superimposed onto the viral capsid (grey) with TECs labelled (**a**). The location of the 10 TECs within the virion is depicted in panel **b** with the poles and tropics labelled. A zoomed image of the boxed area (red) in **a** is shown with the capsid (grey), RdRp and VP4 (blue) and RNA (radially coloured) labelled with TEC-bound RNA highlighted in the dashed box (**c**). The averaged TEC structure is presented in panel **d** showing the capsid (grey), RdRp (purple), VP4 (blue) and RNA (brown). The major and minor grooves in the RNA are indicated by arrows (yellow and white, respectively). Figure adapted from Zhang et al. 2015

Cytoplasmic polyhedrosis virus (CPV) belongs to the *reoviridae* family of viruses and encodes a segmented double-stranded RNA genome encapsidated within a single-shelled capsid. Reoviruses perform *in virio* mRNA synthesis using their genome segments as templates for the transcriptional enzyme complexes (TECs) (Lawton et al. 2000; Xia et al. 2003). Zhang et al. performed icosahedral reconstruction of CPV and subsequently used this to restrain the refinement procedure whilst imposing no symmetry (Zhang et al. 2015). This asymmetric analysis followed by averaging of the TECs revealed that each CPV particle contains 10, one per genome segment. These undergo conformational changes to switch from the quiescent state to the transcriptionally active form. The RNA-dependent RNA polymerase (RdRp), VP4 protein and viral RNA were all resolved in both states as well as TEC interactions with the inner surface of the capsid (see Fig. 4c, d). The 10 TECs were found to have precise locations within the virion with 3 each in the north tropic, north pole and south pole positions as well as a single TEC located in the south tropic (see Fig. 4b) (Zhang et al. 2015). This study was a landmark development in cryoEM, being one of the first to describe determination of macromolecular complexes inside virions with incomplete symmetries/occupancies.

## Asymmetric portal assemblies in viruses

**Fig. 5:**
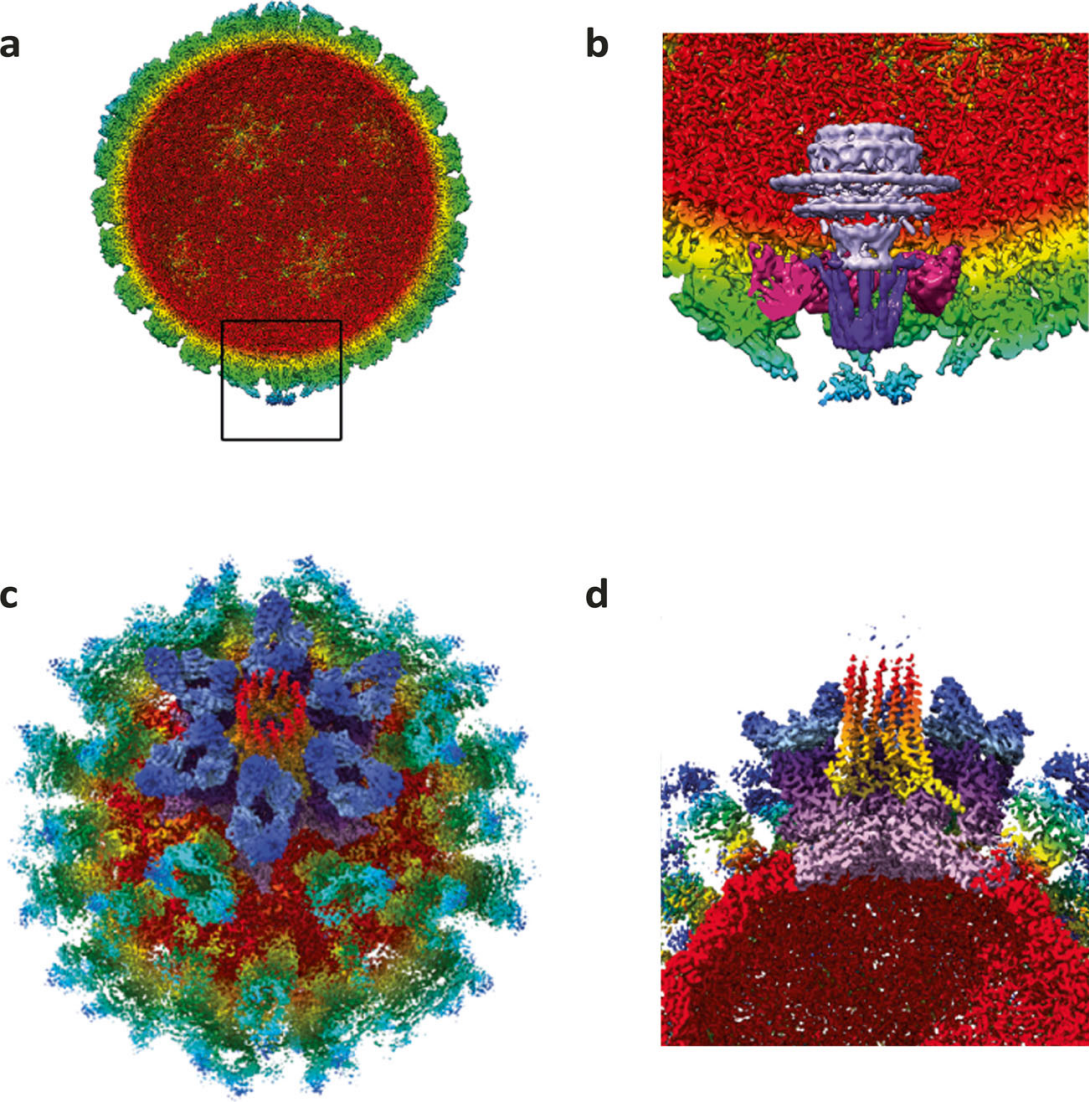
Portals and portal-like assemblies identified using focussed classification. Focussed classification was utilised to identify key features of the HSV1 portal vertex (**a** and **b**). The HSV virion is visualised from the inside and radially coloured with the portal vertex boxed (**a**) and a zoom image shown (**b**; mauve: the portal, purple: portal vertex protein and pink: periportal triplex-like density). The whole FCV virion is shown (**c**) coloured radially and to highlight the receptor proteins (blue) and VP2 portal-like assembly (red). A slice through the receptor decorated FCV virion is shown (**d**) with the VP2 portal-like assembly (yellow to red), assembly proximal VP1 (purple) and receptor (blue) proteins visible within the capsid. Images adapted from Conley et al. 2019 and McElwee et al. 2018 under a creative commons attribution 4.0 international licence

Herpes simplex virus 1 (HSV1) is an enveloped virus of approx. 125 nm in diameter containing a double-stranded DNA genome packaged inside a *T* = 16 icosahedral shell (Dai and Zhou 2018; Yuan et al. 2018). A proteinaceous layer, termed the tegument, lays beneath the viral envelope surrounding the genome containing capsid/nucleocapsid which functions to deliver the viral genome to the nucleus of an infected cell, through the nuclear pore complex (Ojala et al. 2000; Pasdeloup et al. 2009). The site of genome insertion/release from the capsid is at a predetermined 5-fold symmetry axis known as the portal vertex (Newcomb et al. 2001). Focussed classification was employed to resolve features of the portal vertex. Briefly, orientations were determined for each particle assuming icosahedral symmetry. The symmetry of the icosahedral particle was then expanded such that each particle had 60 associated orientations. Relion was then used to perform masked 3D classification using a cylindrical mask to focus on the five-fold axes only (Scheres 2016; Zhou et al. 2015; Mcelwee et al. 2018; Conley et al. 2019). One class demonstrated a structure different to that of the known penton vertex i.e. showed the portal vertex. Upon inspection of the metadata file, most particles contributed 5 views (and thus C5 symmetry was imposed by the data). This method of relaxed symmetry reconstruction allowed the identification of 2 novel features of the HSV1 portal vertex (see Fig. 5a, b) as well as revealing the composition of the portal-vertex-associated tegument (PVAT) which is formed from 10 copies of pUL25 (C-terminal domain). This analysis also revealed that the viral genome is packaged in a highly ordered left-handed spool and that one end of the viral DNA extends through the portal, a feature that has previously only been described for non-eukaryotic viruses (Mcelwee et al. 2018).

Feline calicivirus (FCV) is classified in the *caliciviridae* (alongside the clinically relevant Norovirus) and encodes a single-stranded, positive-sense RNA genome encapsidated within a *T* = 3 icosahedral capsid. The capsid is formed of 180 copies of the major capsid protein VP1 which assemble with dimer-clustering, adopting slightly differing conformations, termed A/B and C/C as for bacteriophage MS2 and Qβ. Upon binding its receptor (feline junctional adhesion molecule A), a conformational change occurs within the capsid resulting in a 15° anti-clockwise rotation of the capsomeres (Conley et al. 2019; Bhella and Goodfellow 2011; Bhella et al. 2008). These conformational changes caused blurring of the density in the protruding regions of the A/B and C/C dimers. To overcome this, focussed classification (Mcelwee et al. 2018; Scheres 2016; Zhou et al. 2015) was used to reveal the different conformations of the A/B and C/C capsomeres. In the course of this analysis, a novel portal-like assembly (formed of 12 copies of the VP2 minor capsid protein) was identified at a unique 3-fold symmetry axis (see Fig. 5c, d). The conformational changes observed in VP1 allow for the extrusion of the previously encapsidated VP2 proteins as well as the formation of a small pore in the capsid shell. It is hypothesised that the VP2 portal-like assembly functions as a method of endosomal escape and genome delivery, a process not well understood in positive-sense RNA containing viruses such as the *caliciviridae* (Conley et al. 2019).

## Summary

An increasing number of asymmetric 3D analysis methods for cryoEM data sets are emerging which are resulting in the discovery of novel capsid features. Basic asymmetric analysis (imposing no symmetry) has become commonplace in the cryoEM field with other methods such as focussed classification and localised reconstruction being increasingly adopted. These techniques are informing advancements in the field of virology with both known structures being better understood e.g. rotavirus spike occupancy (Ilca et al. 2015) as well as the identification of new structures previously unseen with icosahedral reconstruction, e.g. FCV portal-like assembly (Conley et al. 2019).
